# Colouterine fistula after polymyomectomy: a case report

**DOI:** 10.1186/1752-1947-8-199

**Published:** 2014-06-18

**Authors:** Jennifer Uzan, Martin Koskas, Pierre Fournier, Anne Laure Margulies, Dominique Luton, Chadi Yazbeck

**Affiliations:** 1 Obstetrics and Gynecology Department, Bichat-Claude Bernard Hospital. APHP, Paris 7 University, 46 rue Henri Huchard, 75018 Paris, France; 2 General Surgery Department, Bichat-Claude Bernard Hospital. APHP, Paris 7 University, 46 rue Henri Huchard, 75018 Paris, France; 3 Obstetrics, Gynecology and Reproductive Medicine Department, Bichat-Claude Bernard University Hospital, 46, rue Henri Huchard, 75018 Paris, France

**Keywords:** Colouterine fistula, Fertility preservation, Myomectomy, Surgical management

## Abstract

**Introduction:**

Colouterine fistula is a very rare condition; most cases described in the literature are secondary to complications of diverticulitis in elderly patients.

**Case presentation:**

We report the case of a 34-year-old African woman who presented with a colouterine fistula secondary to polymyomectomy, which was diagnosed in the setting of severe endometritis. She had a Hartmann procedure and abundant irrigation of her abdominal and uterine cavities followed by placement of a double drainage in order to preserve fertility. This is the first case of a conservative management of the uterus in such conditions.

**Conclusion:**

Conservative surgery in colouterine fistula should be discussed as an alternative to hysterectomy in young infertile women.

## Introduction

Colouterine fistula is a very rare condition; most cases described in the literature occurred secondary to complications of diverticulitis in the elderly [1,2]. Other circumstances include sigmoid malignancy [3], radiotherapy and iatrogenic conditions such as insertion of intrauterine devices [4], endometrial curettage with uterine and bowel perforation [5], or obstetrical injury [6].

## Case presentation

We report the case of a 34-year-old infertile African woman with no medical or surgical history. She was referred to our hospital for intense abdominal pain associated with sepsis and fecaloid leukorrhea.

**Figure 1:**
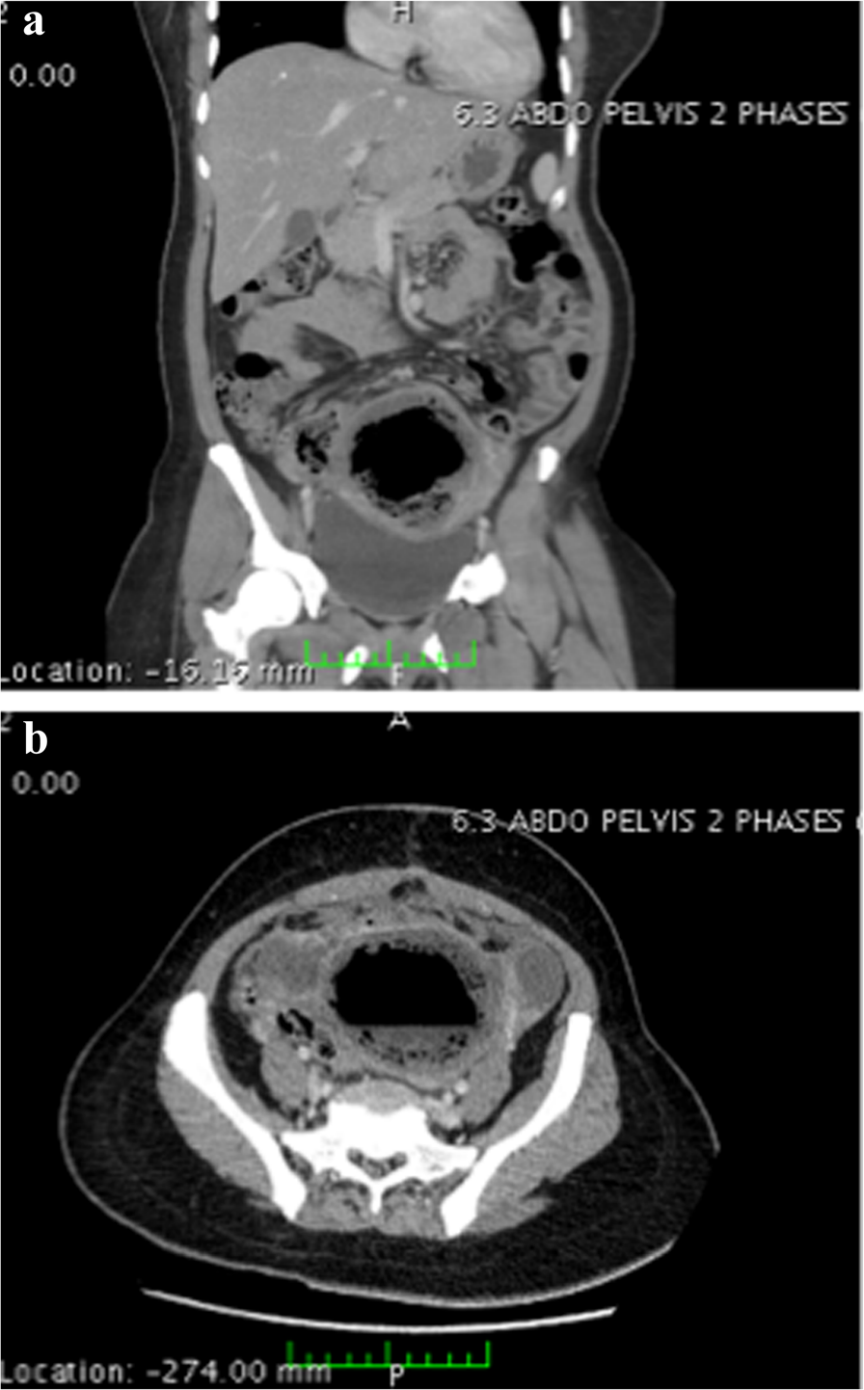
**Abdominal computed tomography scan revealing hydroaeric levels inside the uterus. a**: coronal view. **b**: transverse view.

She had a surgical management of a symptomatic leiomyomatous uterus 20 days earlier in another clinic: preoperative selective uterine artery embolization (UAE) of both her uterine arteries was immediately followed by median laparotomy and ablation of a posterior interstitial leiomyoma of 70mm and an anterior subserous leiomyoma of 45mm; two hysterotomies were made to enucleate the leiomyomas and then sutured with absorbable thread (Vicryl®), and an anti-adhesion membrane (Seprafilm®) with epiploon interposition was used on her uterine sutures.Emergency biological tests revealed a C-reactive protein of 300mg/L. An abdominal computed tomography (CT) scan showed intrauterine hydroaeric levels, which confirmed the diagnosis of a colouterine fistula (Figure 1a and 1b).

Intravenous antibiotics were started immediately (third generation cephalosporins, metronidazole and gentamicin) and she was counseled about the risk of hysterectomy but she insisted on trying a conservative management.

An immediate laparotomy enabled us to isolate the fistula between her sigmoid and her uterine fundus, with feces inside her uterus associated with several lesions of myometritis but with no sign of fecal peritonitis; Hartmann’s procedure involved resection of 15cm of her sigmoid colon with creation of a protective abdominal colostomy, and was followed by abundant irrigation of her abdominal and uterine cavities; the uterine edge of the fistula was estimated to have a diameter of 8mm. It was not immediately closed, but used to place an irrigation system associated with a cervical drainage of the uterus. A drainage of her abdominal cavity was also placed. Antibiotic therapy was reinforced with Tazocilline® (piperacillin-tazobactam) and amikacin. Irrigation was stopped at day 3 and drainage removed at day 5 after surgery.

**Figure 2:**
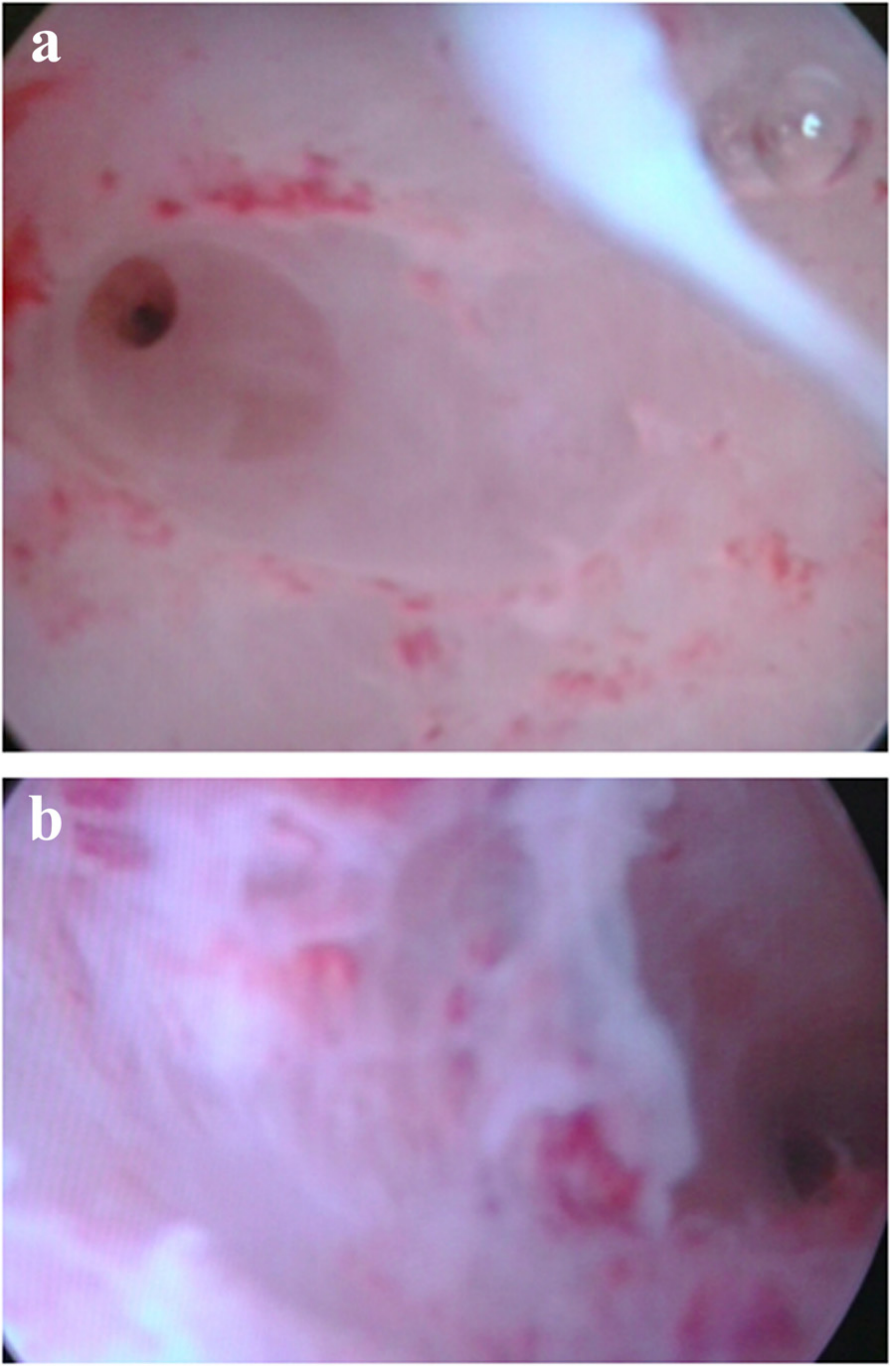
**Postoperative hysteroscopic findings. a**: right tubal ostium. **b**: left tubal ostium.

Histologic analysis did not show malignancy. She was then seen monthly for clinical examination and pelvic ultrasound by a referent gynecologist.At 3 months a pelvic ultrasound showed a heterogeneous myometrium, endometrial atrophy and a small uterine cavity. Her digestive continuity was reestablished at 5 months by the referent surgeon. At 12 months, she was still amenorrheic despite combined hormonal treatment (ethinylestradiol 0.03mg + levonorgestrel 0.15mg). Operative hysteroscopy showed a cavity length of 80mm, endometrial atrophy, and a fundal synechia that reduced her uterine cavity volume and prevented the visibility of both tubal ostia. The synechia was removed under ultrasound guidance (Figure 2a and 2b). At latest news, menstrual cycles partially resumed 3 months later, but she did not become pregnant because of other personal problems.

## Discussion

To the best of our knowledge, this is the first case of colouterine fistula after polymyomectomy, conservatively managed in an emergency setting. The two main etiologic hypotheses could be: (i) preoperative UAE usually associated with reduced blood supply, which could interfere with myometrial cicatrization and increase the risk of uterine fistula [7-9]; and (ii) the use of anti-adhesion barrier (Seprafilm®) on uterine sutures with epiploon interposition could have connected the uterine suture to the bowel leading to fistula formation. Such practice has been suspected to increase leak related events (fistula, abscess, peritonitis and sepsis) in a multicenter randomized study comparing complications of abdominal surgery with and without the use of Seprafilm® [10].

Most of the 24 cases of colouterine fistulas reported in the English and French literature were due to diverticulitis. Classically, they affected older patients for whom a concomitant subtotal or total hysterectomy was the preferred therapeutic choice [1,11]. Uterine conservation in young patients was reported in rare cases (Table 1): excision of myometrium surrounding the fistula and reparation of the uterine wall associated with restitution of sigmoid defect with or without drainage [12-15]. It is important to mention that all these cases were not managed in an emergency setting with sepsis [16], except for one case of a pregnant patient who had a subtotal hysterectomy associated with bowel resection [17].

**Table 1:** Characteristics of patients treated conservatively for colouterine fistulas

**Author (year)**	**Age (years)**	**Etiology**	**Emergency setting**	**Surgical management**	**Fertility at late follow up**
Choi (2012) [12]	81	Diverticulitis	Yes	Bowel resection	No
Zeino *et al*. (2011) [13]	43	Intrauterine device	No	Direct repair (suture)	No
Dadhwal *et al*. (2008) [14]	24	Foreign body in the uterus	No	Direct repair (suture)	No
Shaw *et al*. (1995) [15]	26	Foreign body ingestion	No	Bowel resection	?
Fadel (1977) [16]	32	Myomectomy	No	Bowel resection	?

Fertility of women treated conservatively for a colouterine fistula was never reported. Endometrial thickness should be monitored and patients should have a regular follow up.

## Conclusions

Colouterine fistula following myomectomy is a very rare condition in young women. The diagnosis is easily made with clinical history and abdominal CT scanning. Risk factors might include preoperative UAE, which should be used with caution before myomectomy. Conservative surgical management is challenging in case of severe infection, but should be discussed if fertility preservation is strongly desired. The therapeutic management combines intravenous large spectrum antibiotics and surgery and has two objectives: treat the fistula and its gynecological consequences taking into account the preservation of fertility. Despite a favorable outcome, fertility prognosis is still to be assessed.

## Consent

Written informed consent was obtained from the patient for publication of this case report and accompanying images. A copy of the written consent is available for review by the Editor-in-Chief of this journal.

## Abbreviations

CT: Computed tomography; UAE: Uterine artery embolization.

## Competing interests

The authors declare that they have no competing interests.

## Authors’ contributions

JU and CY analyzed and interpreted the patient data regarding the emergency management and the postoperative follow up. PF and CY performed the surgery. JU was a major contributor in writing the manuscript. MK and ALM revised the manuscript. DL recruited and supported the management of the case. All authors read and approved the final manuscript.

## References

[B1] Moquet PY Letoquart JP Pompilio M Kunin N La Gamma A Mambrini A Sigmoido-uterine fistula of diverticular origin. Review of the literature a propos of a case J Chir (Paris) 1991 128 419 423 1761590

[B2] Sentilhes L Foulatier O Verspyck E Roman H Scotte M Marpeau L Colouterine fistula complicating diverticulitis: a case report and review of the literature Eur J Obstet Gynecol Reprod Biol 2003 110 107 110 10.1016/s0301-2115(03)00086-1 12932884

[B3] Halevy A Bracha M Jeroukhimov I Schneider D Nesterenko V En bloc resection for malignant colouterine fistula Tech Coloproctol 2010 14 37 39 10.1007/s10151-009-0555-6 20130950

[B4] Pinto P Sharma L Kini P Chronic utero-rectal fistula with menochezia and amenorrhea Int J Gynaecol Obstet 1990 33 77 78 10.1016/0020-7292(90)90659-9 1974538

[B5] Hawkes SZ Enterouterine fistula; with a review of the literature and report of an unusual case Am J Obstet Gynecol 1946 52 150 153 20992597

[B6] Martin DH Hixson CH Wilson ECJ Enterouterine fistula; review; report of an unusual case Obstet Gynecol 1956 7 466 469 13309921

[B7] Donnez O Jadoul P Squifflet J Donnez J Unusual complication after uterine artery embolization and laparoscopic myomectomy in a woman wishing to preserve future fertility Fertil Steril 2007 2008 90 e5 e9 10.1016/j.fertnstert.2008.05.091 18692795

[B8] Gutierrez LB Bansal AK Hovsepian DM Uteroenteric fistula resulting from fibroid expulsion after uterine fibroid embolization: case report and review of the literature Cardiovasc Intervent Radiol 2012 35 1231 1236 10.1007/s00270-011-0318-4 22159908

[B9] Dewdney SB Mani NB Zuckerman DA Thaker PH Uteroenteric fistula after uterine artery embolization Obstet Gynecol 2011 118 434 436 10.1097/AOG.0b013e31821082a3 21768845

[B10] Beck DE Cohen Z Fleshman JW Kaufman HS van Goor H Wolff BG A prospective, randomized, multicenter, controlled study of the safety of Seprafilm adhesion barrier in abdominopelvic surgery of the intestine Dis Colon Rectum 2003 46 1310 1319 10.1007/s10350-004-6739-2 14530667

[B11] Hoekstra AV Doan T Kosinski A Dini M Colouterine fistulas in elderly women: a report of 2 cases J Reprod Med 2005 50 796 800 16320560

[B12] Choi PW Colouterine fistula caused by diverticulitis of the sigmoid colon J Korean Soc Coloproctol 2012 28 6 321 324 10.3393/jksc.2012.28.6.321 PMC3548148 23346512

[B13] Zeino MY Wietfeldt ED Advani V Ahad S Younkin C Hassan I Laparoscopic removal of a copper intrauterine device from the sigmoid colon JSLS 2011 15 4 568 570 10.4293/108680811X13176785204661 PMC3340974 22643520

[B14] Dadhwal V Ghosh B Jindal VL Vaid A Agarwal S Mittal S A case of colouterine fistula managed laparoscopically J Minim Invasive Gynecol 2008 15 652 654 10.1016/j.jmig.2008.06.014 18722981

[B15] Shaw FM Reinus JF Leikin EL Tejani N Recurrent chorioamnionitis and second-trimester abortion because of an enterouterine fistula Obstet Gynecol 1995 86 639 641 10.1016/0029-7844(95)00125-b 7675395

[B16] Fadel HE Ileo-uterine fistula as a complication of myomectomy. Case report Br J Obstet Gynaecol 1977 84 312 313 10.1111/j.1471-0528.1977.tb12584.x 870014

[B17] Sriganeshan V Willis IH Zarate LA Howard L Robinson MJ Colouterine fistula secondary to endometriosis with associated chorioamnionitis Obstet Gynecol 2006 107 451 453 10.1097/01.AOG.0000168443.22820.2e 16449145

